# RNase L Cleavage Products Promote Switch from Autophagy to Apoptosis by Caspase-Mediated Cleavage of Beclin-1

**DOI:** 10.3390/ijms160817611

**Published:** 2015-07-31

**Authors:** Mohammad Adnan Siddiqui, Sushovita Mukherjee, Praveen Manivannan, Krishnamurthy Malathi

**Affiliations:** Department of Biological Sciences, 2801 W. Bancroft St., University of Toledo, Toledo, OH 43606, USA; E-Mails: Mohammad.Siddiqui@rockets.utoledo.edu (M.A.S.); sushovitam@yahoo.com (S.M.); Praveen.Manivannan@rockets.utoledo.edu (P.M.)

**Keywords:** autophagy, apoptosis, RNase L, dsRNA, Beclin-1

## Abstract

Autophagy and apoptosis share regulatory molecules enabling crosstalk in pathways that affect cellular homeostasis including response to viral infections and survival of tumor cells. Ribonuclease L (RNase L) is an antiviral endonuclease that is activated in virus-infected cells and cleaves viral and cellular single-stranded RNAs to produce small double-stranded RNAs with roles in amplifying host responses. Activation of RNase L induces autophagy and apoptosis in many cell types. However, the mechanism by which RNase L mediates crosstalk between these two pathways remains unclear. Here we show that small dsRNAs produced by RNase L promote a switch from autophagy to apoptosis by caspase-mediated cleavage of Beclin-1, terminating autophagy. The caspase 3-cleaved C-terminal fragment of Beclin-1 enhances apoptosis by translocating to the mitochondria along with proapoptotic protein, Bax, and inducing release of cytochrome C to the cytosol. Cleavage of Beclin-1 determines switch to apoptosis since expression of caspase-resistant Beclin-1 inhibits apoptosis and sustains autophagy. Moreover, inhibiting RNase L-induced autophagy promotes cell death and inhibiting apoptosis prolongs autophagy in a cross-inhibitory mechanism. Our results demonstrate a novel role of RNase L generated small RNAs in cross-talk between autophagy and apoptosis that impacts the fate of cells during viral infections and cancer.

## 1. Introduction

Autophagy and apoptosis are interconnected stress response pathways that determine cell fate decisions between survival and death to maintain cellular homeostasis. Apoptosis eliminates cells primarily through activation of caspases, in signaling cascades, to disintegrate cellular content accompanied by nuclear fragmentation, cell shrinkage and membrane blebbing which are characteristic of apoptotic cell death pathways. Macroautophagy (referred to as autophagy hereafter) facilitates recycling of long-lived proteins, damaged or surplus organelles and pathogens by sequestering in double-membraned autophagosomes which fuse with lysosomes for degradation. Although autophagy is a cytoprotective response and may suppress or delay cell death, excessive or prolonged autophagy induces cell death by priming cells to undergo apoptosis. Depending on the cellular context and trigger, autophagy and apoptosis may function simultaneously and in other instances the cell switches between the two responses that are mutually inhibitory suggesting complex interconnections and shared regulators between the two pathways [1,2].

Cross-regulation of autophagy and apoptosis relies on proteins that are shared and many of the autophagy proteins serve as molecular switches between autophagy and apoptosis. Beclin1, a BH3-only protein, is a key regulator of autophagy [3]. Association of Beclin1 with lipid kinase III (Vps34) is essential for initiation of autophagosome formation [4]. Several cofactors (Atg14L, UVRAG, Bif-1, Rubicon, Ambra1, HMGB1, VMP1, IP3R, PINK and Survivin) regulate the activity of Beclin1-Vps34 core complex to induce autophagy [5,6]. Autophagy is inhibited by binding of Bcl2 or Bcl-xL with the BH3 domain of Beclin1, which interferes with Beclin1-Vps34 complex formation [6,7,8,9]. Bcl2 also binds to Atg12 to promote mitochondrial apoptosis [10]. Nutrient starvation and other stress conditions, including RNase L activity, dissociate Beclin1-Bcl2 complex, allowing Beclin1 to form a core complex with Vps34 and stimulate autophagy [11]. The cross-talk of autophagy and apoptosis could be regulated by JNK activity. Phosphorylation of Bcl2 by c-jun N-terminal kinase (JNK) triggers release from Beclin1 [9,12,13]. Alternately, Beclin1 is phosphorylated by death associated protein kinase (DAPK) in the BH3 domain, which reduces its affinity for Bcl-xL [14]. Disruption of Beclin1-Bcl2 or Bcl-xL complex is an early step in autophagy induction and also serves as a convergent point for autophagic and apoptotic machinery. Beclin1, Atg4D and Vps34 are cleaved by caspase-3 or caspase-8 to inhibit autophagy and promote cell death, and autophagy degrades active caspase-8 [15,16,17,18,19,20]. FLIP inhibits caspase-8 activity and competes with LC3 for binding ATG3 to inhibit autophagy [21]. Atg5 conjugates to Atg12 and associates with isolation membrane to form autophagosomes along with other Atg proteins [22]. Proteolytic cleavage of Atg5 by calpain generates truncated Atg5 which translocates to mitochondria to induce apoptosis [23]. Caspase-9 directly interacts with Atg7 and facilitates formation of autophagosomes and Atg7 represses the apoptotic activity of caspase-9 [24]. The physical and functional interaction between autophagy and apoptotic proteins form the basis for crosstalk and modulation of these pathways dictate the outcome in response to specific triggers.

Virus infection of cells induces secretion of type I interferons (IFNs) which act in autocrine and paracrine ways to induce expression of interferon-stimulated-genes (ISGs) with antiviral, antiproliferative and apoptotic functions [25]. Viral nucleic acids serve as pathogen associated molecular patterns (PAMPs) and include double-stranded RNAs produced during viral infections that are sensed by pathogen recognition receptors (PRRs) and initiate innate immune signaling pathways to stimulate IFN production [26,27]. The PAMPs could be viral genomes, replicative intermediates, annealed viral RNA of opposite strands and RNAs with secondary structures. The cytosolic Rig-like helicases (RLHs), endosomal Toll-like receptors (TLR), dsRNA-dependent protein kinase R (PKR) and Oligoadenylate synthetases (OAS) all serve as dsRNA sensors [28]. Double-stranded RNA signals through PKR and Rig-I to induce apoptosis in a cell type dependent manner [29,30,31,32,33,34,35]. Short dsRNA (23–24 nt) induced apoptosis involving PKR and p38 in human granulosa tumors [34]. During SeV infection, Rig-I activates IRF3 to bind with Bax and translocate to the mitochondria to induce apoptosis, and activation of IRF3 through TLR3 and PKR did not cause apoptosis [36]. Induction of apoptosis in response to dsRNA depends on cell type, size of dsRNAs and expression proapoptotic and antiapoptotic proteins [37]. The 2′-5′-oligoadenylate synthetase (OAS)/Ribonuclease L (RNaseL) system is an antiviral pathway that is activated in virus-infected cells or in response to IFN [38]. OAS proteins are activated by viral dsRNA PAMPs or RNAs with secondary structures to produce 2–5A [p*_x_*5′A(2′p5′A)*_n_*; *x* = 1–3; *n* ≥ 2] from cellular ATP, which in turn binds specifically to the latent endoribonuclease, RNase L [39]. 2-5A binding promotes dimerization of RNase L and converts it to an active enzyme. Activated RNase L cleaves single stranded viral and host RNAs including 18S and 28S rRNA to mediate direct antiviral effects [40,41]. Activity of RNase L generates small RNA with duplex structures which initiates signaling events through Rig-I-like helicases, Rig-I and MDA5 to amplify the production of IFNβ [42]. In addition, the RNA cleavage products stimulate inflammasome activation by binding to DExD/H helicase, DHX33 [43]. Activation of RNase L induces apoptosis involving activity of caspase 3 [44,45] in some cell types which correlated with basal levels of OAS and RNase L [46]. We have shown recently that activation of RNase L induces autophagy involving the activities of JNK and PKR [11]. Phosphorylation of Bcl2 by JNK disrupted complex with Beclin-1 and promoted complex formation with Vps34 which is required for autophagosome formation. In this study we explored how RNase L induces autophagy and apoptosis and the role in crosstalk between these two pathways. Many viruses directly impact autophagic and apoptotic pathways and to study the unique contribution of RNase L without the complications of viral proteins, we have used 2–5A to directly activate RNase L or dsRNA to activate OAS1 to produce endogenous 2–5A to study the effect on regulation of autophagy and apoptosis. Our results show that activation of RNase L induces autophagy as we have demonstrated previously [11] and the small dsRNAs generated by RNase L enzyme activity promote a switch from autophagy to apoptosis by caspase-mediated cleavage of Beclin-1. Cleavage of Beclin-1 is an important determinant of switch from autophagy to apoptosis and inhibiting RNase L induced autophagy accelerates cell death by apoptosis. Our studies identify a novel role for RNase L-cleaved RNAs in regulating switch from autophagy to apoptosis that can determine fate of cells during viral infections.

## 2. Results

### 2.1. RNase L and dsRNA Signaling Pathways Regulate Cross-Talk between Autophagy and Apoptosis

**Figure 1:**
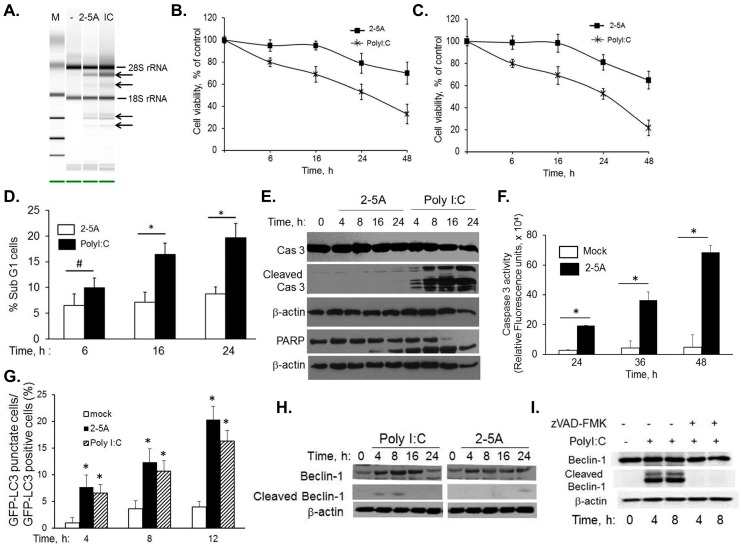
Cleavage of Beclin-1 in RNase L-mediated cross-talk between autophagy and apoptosis. HT1080 cells were transfected with 10 µM of 2–5A or 2 µg/mL of PolyI:C and (**A**) RNase L-mediated cleavage of rRNA (arrows) was analyzed on RNA chips using the Agilent Bioanalyzer 2100 after 6 h. Cell viability was determined at indicated times by (**B**) MTT colorimetric assays, (**C**) trypan blue dye exclusion assay normalized to control cells or (**D**) uptake of PI by dying cells as measured by flow cytometry after staining with PI. Results are representative of three independent experiments performed in triplicate ± SD; (**E**) Cleavage of Caspase 3 and PARP in cell lysates from 2–5A or PolyI:C transfected cells was analyzed on immunoblots and normalized to β-actin levels; (**F**) Caspase 3/7 activity was measured in 2–5A transfected cells at indicated times using rhodamine-labeled caspase-3 and -7 substrate (ApoONE homogenous caspase-3 and -7 assay kit (Promega). Results are representative of three independent experiments performed in triplicate ± SD; (**G**) GFP-LC3 expressing HT1080 cells were mock treated, transfected with 10 µM of 2–5A or 2 µg/mL of PolyI:C for indicated times and the percentage of GFP^+^ cells showing puncta formation compared to mock treated cells was analyzed. Results shown represent mean ± SEM for three experiments and at least 100 cells were analyzed per assay, *p* values are shown as compared with mock treated cells; (**H**) Cleavage of Beclin-1 was monitored in response to 2–5A or PolyI:C for indicated times on immunoblots and normalized to β-actin levels; (**I**) HT1080 cells expressing Flag-Beclin-1 were pretreated with zVAD-FMK (20 µM) or not for 1 h followed by 2 µg/mL of PolyI:C for indicated times. Cleavage of Beclin-1 was determined on immunoblots and normalized to β-actin levels. Results are representative of three independent experiments. Student’s *t* test was used to determine *p* values. * *p* < 0.001, # *p* < 0.05.

HT1080 cells were transfected with 2–5A or synthetic dsRNA, polyI:C, to determine the role of RNase L in autophagy and apoptosis. Activation of RNase L in intact cells was monitored by transfecting 2–5A or polyI:C and detecting specific cleavage products of 18S and 28S rRNA on RNA chips and analyzed using Agilent Bioanalyzer (Figure 1A) [47,48]. Effect of activation of RNase L on cell viability was determined by MTT assay and trypan blue exclusion (Figure 1B,C). Cells treated with 2–5A did not show any difference in cell viability until 16 h; 65% cells remained viable at 48 h. In contrast, polyI:C reduced cell viability progressively with time; less than 30% or 17% cells were viable at 48 h. After transfection of HT1080 cells with 2–5A or polyI:C for indicated times, the percentage of sub G1 cells, which represent apoptotic cells, was quantified by propidium iodide (PI) staining and flow cytometry. Significant increase in apoptosis was observed in polyI:C treated cells at 16 and 24 h compared to 2–5A treated samples (Figure 1D). In contrast with 2–5A, caspase 3 cleavage corresponding to cell death was observed in immunoblots starting at 4h in polyI:C treated cells confirming involvement of mitochondrial pathway of apoptosis (Figure 1E). The observed caspase 3 cleavage also correlated with cleavage of PARP, another hallmark of apoptosis. In 2–5A treated cells we observed cell death after 24 h which increased progressively until 48h and this correlated with induction of caspase3/7 activity (Figure 1F). To determine if autophagy is induced simultaneously as apoptosis in cells treated with polyI:C, HT1080 cells were transfected with GFP-LC3 plasmid and 24 h later with 2–5A or polyI:C. The redistribution of GFP-LC3 from diffuse to a punctuate pattern representing autophagosomes (Figure S1) was quantified in 100 cells per field and normalized to GFP^+^ cells. The number of autophagic cells did not vary significantly in cells treated with 2–5A compared to polyI:C to 12 h (Figure 1G). At later time points increased cell death was observed in polyI:C treated cells.

Several studies have shown that autophagy and apoptosis may exist simultaneously or the cells may switch between the two outcomes depending on the signaling pathways activated [1]. For instance, autophagy proteins like Beclin-1, Atg5 and Vps34 are cleaved by caspases or calpains to terminate autophagy and switch to apoptosis. To determine if Beclin-1 is cleaved by caspases, HT1080 cells were transfected with 2–5A or PolyI:C for 4 to 24 h. Cell lysates were subject to immunoblotting using Beclin-1 antibody. Cleavage of Beclin-1 is evident by 4 h in polyI:C treated cells simultaneous with caspase-3 activation (Figure 1H). In comparison, weak cleavage of Beclin-1 is observed in 2–5A-treated cells at 24 h (Figure 1H). Further, inhibiting caspase activity by pretreating cells with caspase inhibitor, zVAD-fmk, prevented cleavage of Flag-Beclin-1, indicating the involvement of caspase in cleavage of Beclin-1 (Figure 1I). Taken together, these results suggest that Beclin-1 is cleaved by activity of caspases which are activated early (4 h onwards) in cells treated with dsRNA and at later timepoints (24 h and later) in cells treated with 2–5A which correlates with onset of apoptosis.

**Figure 2:**
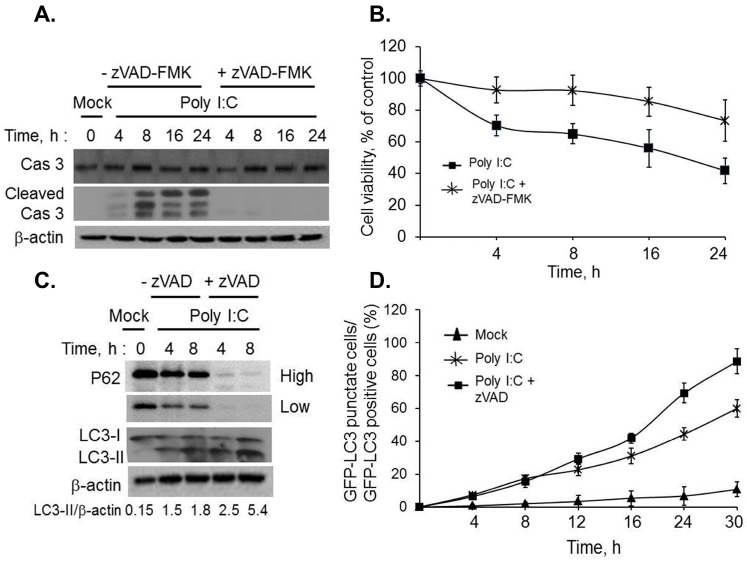
Regulation of autophagy by inhibiting dsRNA-induced apoptosis. HT1080 cells were pretreated with zVAD-FMK (20 µM) or not for 1h followed by 2 µg/mL of PolyI:C for indicated times. (**A**) Cleavage of caspase 3 was determined on immunoblots and normalized to β-actin levels; (**B**) cell viability was determined by MTT assay and normalized to control cells. Results are representative of three independent experiments performed in triplicate ± SD; (**C**) Conversion of unconjugated LC3-I to lipidated LC3-II and degradation of p62 was monitored on immunoblots and normalized to β-actin levels. Band intensity was calculated using Image J software and ratios of LC3-II/β-actin was determined. Results are representative of three independent experiments; (**D**) GFP-LC3 expressing HT1080 cells were pretreated with zVAD-FMK (20 µM) or not for 1 h followed by 2 µg/mL of PolyI:C or mock treated for indicated times. The percentage of GFP^+^ cells showing puncta formation compared to mock treated cells was analyzed. Results shown represent mean ± SEM for three experiments and at least 100 cells were analyzed per assay.

We demonstrated that activation of RNase L induces both autophagy and apoptosis and cleavage of Beclin-1 correlates with caspase activation in response to dsRNA (Figure 1). Next, we investigated if inhibition of apoptosis sustains autophagy and promotes cell survival by preventing cleavage of Beclin-1. HT1080 cells were pretreated or not with caspase inhibitor, zVAD-fmk, followed by polyI:C transfection and inhibition of caspase activity was confirmed by immunoblot analysis (Figure 2A). Significant increase in cell viability was observed by MTT assay (45% to 75%) in cells pretreated with caspase inhibitor followed by dsRNA transfection (Figure 2B). To investigate if inhibiting cell death prolonged autophagy, we monitored conversion of LC3-I to LC3-II on immunoblots and degradation of P62, both are indicators of autophagy (Figure 2C). Further, GFP-LC3 fluorescence was used to microscopically monitor autophagy in cells pretreated with caspase inhibitor followed by polyI:C transfection and compared to polyI:C treated cells. The redistribution of GFP-LC3 from diffuse to a punctuate pattern representing autophagosomes was significantly more in inhibitor treated cells (68% of GFP^+^ cells) as compared to cells not treated with inhibitor (45% of GFP^+^ cells) followed by dsRNA, polyI:C (Figure 2D). Cells not treated with polyI:C did not induce autophagy. Taken together, our data demonstrate that RNase L and dsRNA signaling pathways activated by polyI:C participate in crosstalk between autophagy and apoptosis by caspase-mediated cleavage of Beclin-1.

### 2.2. Inhibiting RNase L-Induced Autophagy Leads to Apoptosis by Cleavage of Beclin-1

**Figure 3:**
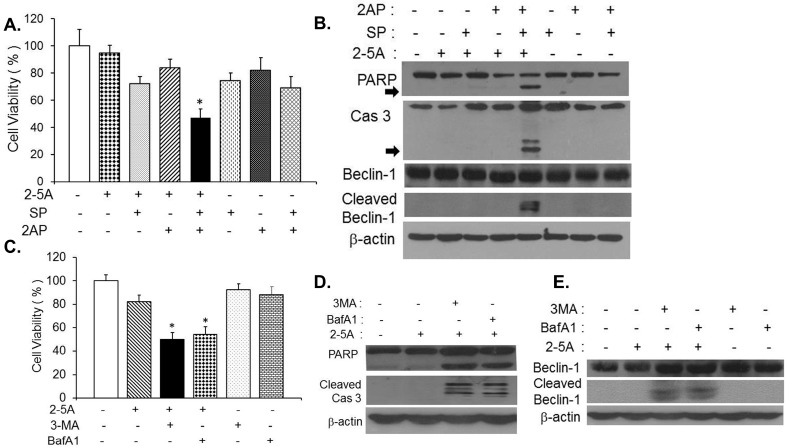
Inhibiting RNase L-induced autophagy activates caspase 3 to cleave Beclin-1. HT1080 cells were pretreated with PKR inhibitor (2-aminopurine, 5 mM), JNK inhibitor (SP600125, 25 µM) or with 2AP and SP600125 combined for 1h prior to 2–5A transfection (10 µM) for 8 h. (**A**) Cell viability was determined by MTT assay. Student’s *t* test was used to determine *p* values of cells treated with 2–5A alone or combined with single inhibitors and inhibitors alone (without 2–5A) and compared to cells treated with 2–5A (10 µM) and 2-amionopurine and SP600125 combined, * *p* < 0.001. Results are representative of three independent experiments performed in triplicate ± SD; (**B**) Cleavage of PARP, Caspase 3 (cleavage products are indicated by arrows) and Beclin-1 was monitored in cell lysates by immunoblotting and normalized to β-actin levels. HT1080 cells were pretreated with autophagy inhibitors, 3-methyladenine (3-MA, 5 mM) or Bafilomycin A1 (Baf A1, 100 nM) for 1 h, followed by 2–5A transfection (10 µM) for 24 h; (**C**) Cell viability was determined by MTT assay after 24 h. Student’s *t* test was used to determine *p* values of cells treated with 2–5A alone or inhibitor alone (without 2–5A) and compared to cells treated with 2–5A and 3-MA or BafA1, * *p* < 0.001. Results are representative of three independent experiments performed in triplicate ± SD; (**D**) Cleavage of PARP, Caspase 3 and (**E**) Beclin-1 was monitored in cell lysates by immunoblotting and normalized to β-actin levels. Results are representative of three independent experiments.

RNase L induces autophagy involving the activities of JNK and PKR as demonstrated previously [11] and we observed induction of caspase 3 which correlated with cleavage of Beclin-1 and apoptosis after 24 h (Figure 1). We first tested if inhibiting RNase L-induced autophagy leads to apoptosis in HT1080 cells transfected with 2–5A. Autophagy was inhibited using pharmacological inhibitors of RNase L-induced autophagy, conventional autophagy inhibitors, gene knock-out or knock-down cells for autophagy genes. HT1080 cells were pretreated with SP600125 (a selective JNK inhibitor), 2-aminopurine (2AP), inhibitor of PKR activity or both followed by transfection with 2–5A to activate RNase L. Treatment of 2–5A transfected cells with both SP600125 and 2-aminopurine reduced cell viability significantly (45% of control) compared to either SP600125 (72% of control) or 2-aminopurine alone (85% of control) (Figure 3A). Consistent with loss of cell viability, cleavage of PARP and caspase 3 was detected on immunoblots in cell lysates with combined inhibitor treatments prior to 2–5A transfection (Figure 3B). Pretreatment of HT1080 cells with 3-methyladenine (3-MA), a class III PI3K inhibitor, that inhibits formation of autophagosomes or Bafilomycin A1 (BafA1), inhibitor of autophagosome/lysosome fusion, prior to 2–5A transfection, similarly, reduced cell viability (46% and 52% respectively) (Figure 3C) along with cleavage of PARP and caspase 3 in cell lysates (Figure 3D). No cleavage of PARP or caspase 3 was observed in cells pretreated with inhibitors alone (data not shown). Our data shows that cleavage of Beclin-1 occurred simultaneously with caspase 3 activation and onset of apoptosis in dsRNA, polyI:C, treated cells. Next we investigated if inhibiting RNase L-induced autophagy resulted in caspase 3 mediated cleavage of Beclin-1 which could trigger apoptosis. Cleavage of Beclin-1 was observed in lysates of cells where RNase L-induced autophagy was inhibited using JNK and PKR inhibitor combined or when cells were pretreated with autophagy inhibitors, 3-MA or Bafilomycin A1 (Figure 3B,E).

**Figure 4:**
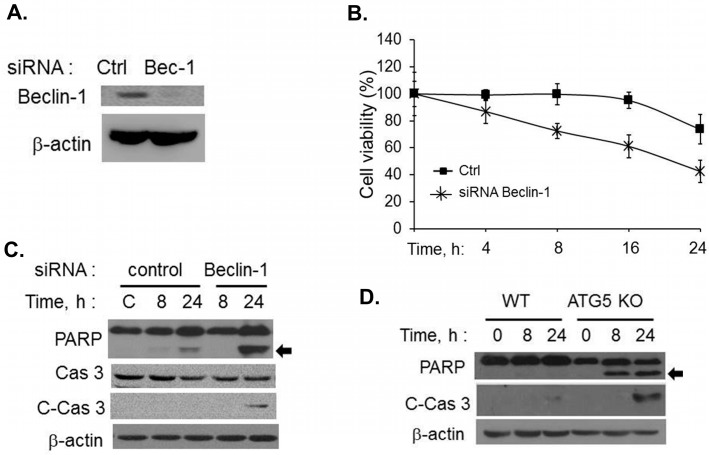
Cells lacking autophagy proteins undergo apoptosis in response to RNase L activation. (**A**) HT1080 cells were transfected with Beclin siRNAs (20 nM) or control siRNAs (20 nM) and knock down of Beclin-1 protein levels were determined on immunoblots; (**B**) Control or Beclin-1 siRNA expressing cells were transfected with 2–5A (10 µM) and cell viability was determined using MTT assay at indicated times. Results are representative of three independent experiments performed in triplicate ± SD; (**C**) Cleavage of PARP (indicated by arrow) and caspase 3 was monitored in cell lysates of 2–5A treated knock-down cells and compared to control cells. Protein levels were and normalized to β-actin; (**D**) WT or Atg5 KO MEFs were transfected with 10 µM of 2–5A for indicated times and induction of apoptosis was monitored by cleavage of PARP (indicated by arrow) and cleaved caspase 3 on immunoblots normalized to β-actin levels. Results are representative of three independent experiments.

To further confirm that inability of the cells to undergo autophagy following activation of RNase L resulted in apoptosis, we knocked down levels of Beclin-1, a protein required for nucleation of autophagosomes, in HT1080 cells using siRNA (Figure 4A) or used primary Mouse Embryonic Fibroblasts (MEFs) lacking ATG5 which is required for autophagosome elongation, and transfected 2–5A to activate RNase L. Activation of RNase L in Beclin-1 knock-down cells reduced cell viability (43%) compared to control cells (73%) and a corresponding cleavage of PARP and caspase 3 was observed (Figure 4B and Figure 4C). Cleavage of caspase 3 and PARP was observed only in MEFs lacking ATG5 and not in WT MEFs transfected with 2–5A (Figure 4D). Our data collectively demonstrates that inhibiting autophagy in RNase L-activated cells by 2–5A treatment leads to apoptosis by caspase-mediated cleavage of Beclin-1.

### 2.3. Caspase-Cleaved Beclin-1 Fragments Induce Apoptosis by Translocation of Bax to the Mitochondria and Release of Cytochrome c

**Figure 5:**
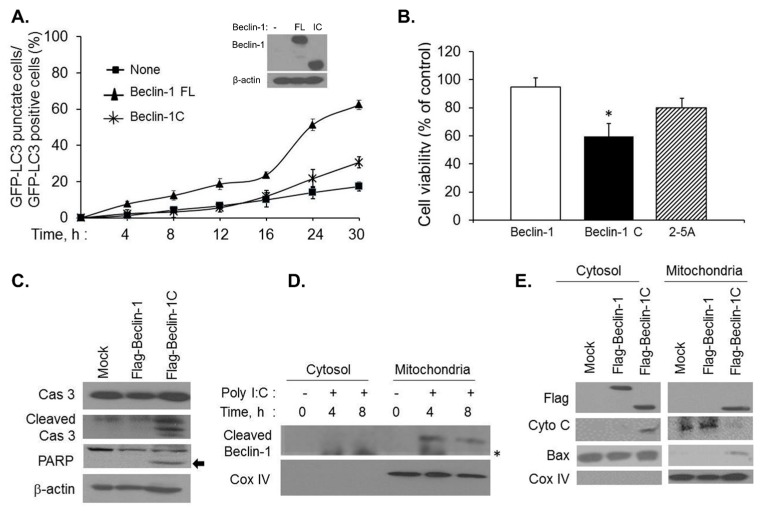
Beclin-1C fragment produced by caspase 3 activity translocates to the mitochondria and enhances apoptosis. (**A**) HT1080 cells were transfected with siRNAs to knock down endogenous Beclin-1 followed by expression of Flag vector (mock), Flag-Beclin-1 (full-length, 1–450) or Flag-Beclin 1C (aa150–450) along with GFP-LC3 plasmid. After 24 h, cells were transfected with 2–5A (10 µM) for indicated times and the percentage of GFP^+^ cells showing puncta formation compared to mock treated cells was analyzed. Immunoblot shows expression of Beclin-1 constructs in knock down cells. Results shown represent mean ± SEM for three experiments and at least 100 cells were analyzed per assay; (**B**) HT1080 cells were transfected with Flag vector (mock), Flag-Beclin-1 (full-length, 1–450) or Flag-Beclin 1C (aa150–450) for 24 h and cell viability was compared to cells transfected with 2–5A (10 µM) for 24 h by MTT assay. Student’s *t* test was used to determine *p* values of cells expressing Beclin-1C and compared to cells expressing Beclin-1 (full length) or cells treated with 2–5A (10 µM), * *p* < 0.001. Results are representative of three independent experiments performed in triplicate ± SD. (**C**) HT1080 cells were transfected with Flag-Beclin-1 (full-length, 1–450) or Flag-Beclin 1C (aa150–450) for 24 h and cleavage of PARP (indicated by arrow) and caspase 3 was monitored on immunoblots of cell lysates and normalized to β-actin levels; (**D**) HT1080 cells were transfected with 2 µg/mL of PolyI:C for 4 or 8 h and cytosolic and mitochondrial fractions were analyzed for cleaved Beclin-1C by immunoblotting. Mitochondrial fractions were marked by CoxIV expression; * non-specific band; (**E**) HT1080 cells were transfected with Flag-Beclin-1 (full-length, 1–450) or Flag-Beclin 1C (aa150–450) for 24 h and cytosolic and mitochondrial fractions were probed with anti-Flag antibodies to detect Beclin-1 full-length and cleaved Beclin-1C fragment, Bax, cytochrome c and CoxIV. Results are representative of three independent experiments.

Beclin-1 is required for autophagy and we observed cleavage of Beclin-1 by caspases in cells where RNase L-induced autophagy is inhibited. These results suggested that apoptosis observed may be mediated by cleaved products of Beclin-1. To determine the impact of Beclin-1 cleavage, we tested if loss of autophagic activity is observed when Beclin-1 is cleaved. We generated Flag-Beclin-1C construct expressing C-terminal 150–450 aminoacid residues or Flag-Beclin-1 (full length, FL) and transfected along with GFP-LC3 construct into HT1080 cells with knockdown of endogenous Beclin-1 using siRNA to the 5ʹUTR of Beclin-1 that does not target Beclin-1 cDNA construct expression (Figure 5A, inset). Punctate expression of GFP-LC3 fluorescence was used to microscopically monitor autophagy in 2–5A transfected cells and quantitated as in Figure 2D. Cells expressing full-length Beclin-1 showed increased autophagy (68%) compared to Beclin-1C expressing cells (32%). Cells with knockdown of endogenous Beclin-1 (none) show significantly reduced autophagy confirming the requirement of Beclin-1 for autophagy (Figure 5A). To further test if the cleaved fragment of Beclin-1 induces apoptosis in the absence of stimulus, HT1080 cells were transfected with Flag-Beclin-1 (full length), Flag-Beclin-1C and compared to 2–5A treatment. Cell viability was measured after 24 h and correlated with cleavage of PARP and caspase 3. Expression of Flag-Beclin-1C decreased cell viability (59%) compared to full-length (94%) or 2–5A treatment (80%) (Figure 5B). Decrease in cell viability that correlated with cleavage of PARP and caspase 3 was observed on immunoblots in cells transfected with Flag-Beclin-1C (Figure 5C). Importantly, Beclin-1C affects cell viability in the absence of additional inducers and in cells that express endogenous Beclin-1. Beclin-1 is a predominantly cytosolic protein and it is likely that the cleaved products have altered cellular localizations in the mitochondria [15,16,49]. To determine if the cleaved Beclin-1 fragment translocated to the mitochondria, HT1080 cells mock treated or treated with PolyI:C and subject to subcellular fractionation to separate cytosolic and mitochondrial fractions. Cleaved fragments of endogenous Beclin-1 were detected in the mitochondrial fraction 4 and 8 h after transfection with PolyI:C as determined by presence of mitochondrial marker, Cox-IV (Figure 5D). To further confirm that C-terminal fragment of Beclin-1 localized to the mitochondria, HT1080 cells were transfected with Flag-Beclin-1 (full-length) or Flag-Beclin-1C and presence of Flag-Beclin-1C was detected in cytosol and mitochondria while the full-length was predominantly present in the cytosol (Figure 5E). Bax is a BH3 domain containing propaoptotic protein which is required for permeabilization of the outermembrane of the mitochondria causing cytochrome C release, which, in turn, induces apoptosis. To determine the physiological consequence of mitochondrial translocation of Beclin-1C in the context of inducing apoptosis, we examined the distribution of proapoptotic proteins like bax and cytochrome C. Expression of Flag-Beclin-1C induced translocation of Bax to the mitochondria along with release of cytochrome C from the mitochondrial membrane into the cytosol (Figure 5E). These results suggest that caspase 3-mediated cleavage of Beclin-1 results in localization of Beclin-1C fragments at the mitochondria along with translocation of Bax and the release of cytochrome C into the cytoplasm inducing apoptosis.

### 2.4. Caspase-Cleavage Resistant Beclin-1 Inhibits Apoptosis Induction and Prolongs Autophagy

**Figure 6:**
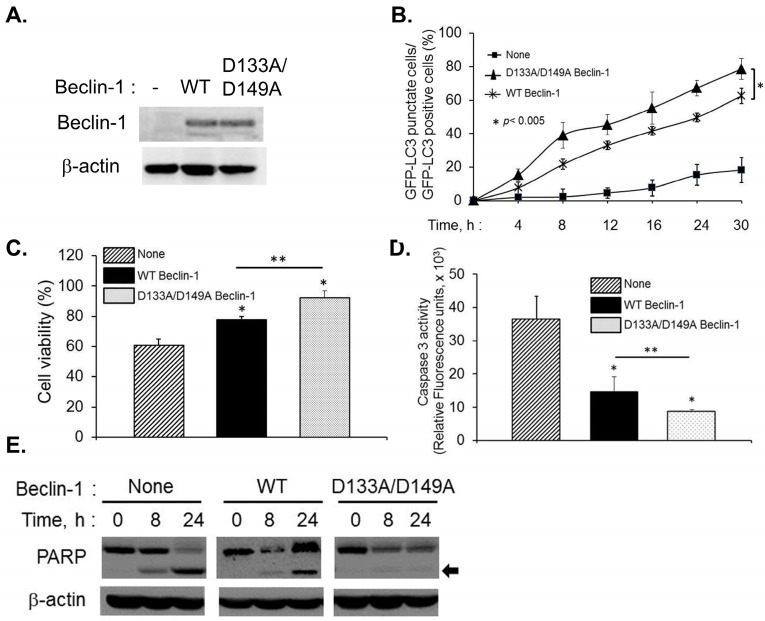
Effect of caspase-resistant Beclin-1 on autophagy and apoptosis in Beclin-1 knock down cells. (**A**) Endogenous Beclin-1 levels were knocked down using siRNA that does not target Beclin-1 cDNA and expression of RNAi-resistant Flag-Beclin-1 (WT) or RNAi and caspase-resistant Flag-Beclin-1 D133A/D149A was detected on immunoblots and normalized to β-actin levels; (**B**) Knock-in cells expressing GFP-LC3 were transfected with 2–5A (10 µM), for indicated times and the percentage of GFP^+^ cells showing puncta formation compared to mock treated cells was analyzed. Student’s *t* test was used to determine *p* values of knock-in cells expressing WT Beclin-1 compared to caspase-resistant Beclin-1. * *p* < 0.005. Results shown represent mean ± SEM for three experiments and at least 100 cells were analyzed per assay; (**C**) Cell viability was quantitated in 2–5A transfected cells using MTT assay and (**D**) Caspase 3 activation was quantitated using rhodamine-labeled caspase-3 and -7 substrate (ApoONE homogenous caspase-3 and -7 assay kit (Promega)). Student’s *t* test was used to determine *p* values of cells expressing Flag-Beclin-1 and compared to cells expressing caspase-resistant Flag-Beclin-1 D133A/D149A Beclin-1 or compared to cells not expressing any Beclin-1 construct. * *p* < 0.001, ** *p* < 0.01. Results are representative of three independent experiments performed in triplicate ± SD; (**E**) Cleavage of PARP (indicated by arrow) was detected in cell lysates of knock-in cells expressing RNAi-resistant Flag-Beclin-1 or RNAi and caspase-resistant Flag-Beclin-1 D133A/D149A transfected with 2–5A to activate RNase L normalized to β-actin levels. Results are representative of three independent experiments.

Our data indicate that Beclin-1 cleavage in RNase L activated cells results in inhibition of autophagy and the translocation of cleaved Beclin-1 fragment, Beclin-1C, to the mitochondria induces apoptosis. Beclin-1 is cleaved by caspase 3 *in vitro* after TDVD^133^ and DQLD^149^ residues and substitution of D133 and D149 completely abolished Beclin-1 cleavage [15,16]. To test the role of caspase 3-mediated cleavage of Beclin-1 on autophagy and apoptosis, we substituted the consensus aspartate residues (D) with alanine (A) at D133 and D149 and analyzed the effect of these caspase-resistant mutations. Endogenous Beclin-1 was knocked down (kd) using siRNA to the 5’UTR of Beclin-1 that does not target Beclin-1 cDNA construct expression. We confirmed knockdown of endogenous Beclin-1 and expression of RNAi resistant Flag-Beclin-1 and Flag-Beclin-1 D133A/D149A on immunoblots (Figure 6A). Formation of autophagosomes monitored by redistribution of GFP-LC3 from diffuse to a punctuate pattern was followed up to 30 h after transfection with –5A. Autophagosome numbers quantitated microscopically in Beclin-1 kd cells receiving no Beclin-1 construct was significantly less (18%) which could be restored by expression of WT Beclin-1 (62%). Furthermore, autophagy induced by caspase-resistant mutant of Beclin-1 was more (79%) than WT expressing cells (Figure 6B). These results are consistent with the observation that WT Beclin-1 can be cleaved by caspase-3 which can promote switch from autophagy to apoptosis and suggest that caspase-resistant Beclin-1 prolongs autophagy in Beclin-1 kd cells following RNase L activation.

To test if the effect of cleavage of Beclin-1 by caspase 3 is the major determinant of inhibition of autophagy and subsequent induction of apoptosis, we tested the effect of activation of RNase L by 2–5A in HT1080 cells with knock-down of Beclin-1 and expressing RNAi-resistant WT Beclin-1 (knock-in) or RNAi and caspase-resistant Beclin-1 (D133A/D149A) on cell viability. Expression of caspase-resistant Beclin-1 in kd cells restored cell viability (92%) compared to WT Beclin-1 (77%) and vector alone (60%) (Figure 6C). Lack of Beclin-1 predictably enhanced cell death in 2–5A treated cells by activation of caspase 3 and cleavage of PARP whereas caspase-resistant Beclin-1 mutant prolonged cell survival and cleavage of PARP and caspase-3 activity was significantly lower (Figure 6D,E). Together, these results suggest that cleavage of Beclin-1 in RNase L activated cells is an important determinant of switch from autophagy to apoptosis.

### 2.5. Small dsRNA Cleavage Products of RNase L Activity Modulate Switch from Autophagy to Apoptosis

**Figure 7:**
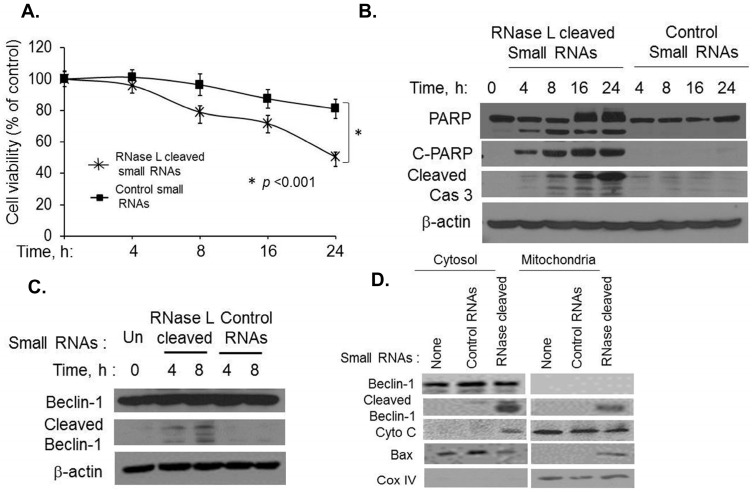
RNase L-generated small RNAs cleave Beclin-1 to promote switch from autophagy to apoptosis. HT1080 cells were transfected with 2 µg/mL of RNase L generated small RNAs or control RNAs (described in methods) for indicated times and (**A**) Cell viability was determined using MTT assay. Results are representative of three independent experiments performed in triplicate ± SD. Student’s *t* test was used to determine *p* values of cells transfected with RNase L generated small RNAs compared to control small RNAs after 24 h. * *p* < 0.001; (**B**) Cleavage of PARP and Caspase 3 was detected in cell lysates using antibodies to PARP, cleaved PARP and Caspase 3; (**C**) Cleavage of Beclin-1 was monitored by immunoblot analysis. β-actin was used as loading control; (**D**) HT1080 cells were transfected with 2 µg/mL of RNase L generated small RNAs or control RNAs for 8 h and cytosolic and mitochondrial fractions were analyzed for cleaved Beclin-1C fragment, Bax, cytochrome c and CoxIV by immunoblotting. Results are representative of three independent experiments.

RNase L is activated in cells by 2–5A or PolyI:C (activates 2’-5’oligoadenylate synthetase to produce 2–5A from cellular ATP) and cleaves single-stranded RNAs to produce RNAs with duplex structures. These small dsRNAs bind to Rig-I-like helicases to produce type I interferon to enhance antiviral responses [42]. Double-stranded RNA is a common product of viral replication and induces apoptosis to eliminate virus infected cells [28]. We explored the possibility that the dsRNA products of RNase L activity behave like synthetic dsRNA, PolyI:C, in inducing apoptosis to curtail autophagy by cleaving Beclin-1. We generated RNase L-cleaved small RNAs from cells transfected with 2–5A and control small RNAs from cells that did not receive 2–5A and purified the RNAs as described previously [42] and in methods. We first tested if transfection of RNase L-cleaved small RNAs into HT1080 cells induced apoptosis in cell viability assays. As shown in Figure 7A, cell death is significantly increased in cells transfected with RNase L-cleaved small RNAs (50% cells are viable) when compared to control small RNAs (81% cells are viable) suggesting that RNase L activity generates RNA products with apoptosis-inducing properties. Treatment of cells with RNase L-cleaved small RNAs resulted in PARP cleavage that increased with time and occurred simultaneously with caspase3 cleavage correlating with increased cell death (Figure 7B). Cleavage of Beclin-1 was observed in PolyI:C transfected cells and at later time points in 2–5A transfected cells (Figure 1F) which had close correspondence with induction of caspase 3. To test if RNase L-cleaved small RNAs induce cleavage of Beclin-1, HT1080 cells were transfected with the respective RNAs and cleavage of Beclin-1 was detected by immunoblotting (Figure 7C). As observed with PolyI:C, cleavage of Beclin-1 was observed starting 4h in cell lysates only in cells that were transfected with RNase L-cleaved small RNAs and not in control RNAs indicating that RNase L generated small RNAs have features of dsRNAs in inducing cell death. We have shown that the C-terminal fragment of Beclin-1 produced by caspase 3 cleavage was localized in the mitochondria along with translocation of Bax which correlated with the release of cytochrome C from the mitochondria to the cytosol (Figure 5E). To investigate if the Beclin-1 cleaved fragments produced by RNase L-cleaved small RNAs also localized to the mitochondria, we performed subcellular fractionation to separate cytosolic and mitochondrial fractions following transfection of cells with RNase L-cleaved or control RNAs. Cells not receiving any RNAs were used as controls. Immunoblot analysis demonstrates the presence of cleaved Beclin-1 only in cells transfected with RNase L-cleaved RNAs and a fraction of the cleaved Beclin-1 was enriched in the mitochondrial fraction as detected with mitochondrial marker, CoxIV (Figure 7D). Furthermore, translocation of Beclin fragments coincided with Bax localization in the mitochondria and the release of cytochrome C to the cytosol (Figure 7D). Our combined results demonstrate that while activation of RNase L induces autophagy as we have published previously [42], the small RNA cleavage products of RNase L activity promote a switch from autophagy to apoptosis by cleaving Beclin-1 to inhibit autophagy and simultaneously the cleaved Beclin-1 fragments translocate to the mitochondria along with Bax and promote release of proapoptotic factors like cytochrome C to enhance apoptosis.

## 3. Discussion

Our results show that RNase L participates in cross-talk between autophagy and apoptosis. RNase L activation has been shown to induce autophagy and apoptosis in many cell types; however it is not clear if these processes occur simultaneously, sequentially or are mutually exclusive. Activation of RNase L induces autophagy as we have demonstrated previously [11] and we show here that the small dsRNAs generated by RNase L enzyme activity promote a switch from autophagy to apoptosis by caspase-mediated cleavage of Beclin-1. Cleavage of Beclin-1 inhibits autophagy and the cleaved C-terminal fragment of Beclin-1 translocates to the mitochondria along with proapoptotic protein, Bax, causing release of cytochrome C to the cytosol inducing apoptosis. Cleavage of Beclin-1 represents a critical step in inducing apoptosis by RNase L since caspase-resistant Beclin-1 mutants inhibit apoptosis and sustain autophagy. Further, inhibiting RNase L-induced autophagy accelerates cell death and inhibiting apoptosis prolongs autophagy. Autophagy and apoptosis are triggered during virus infections and may have antiviral or proviral roles in the pathogenesis of different viruses. These results identify a novel role of RNase L generated RNAs in modulating autophagic and apoptotic pathways which can determine the fate of host cells during virus infections.

Accumulation of dsRNA produced during virus replication and overproduction of viral proteins trigger cell stress and induces programmed cell death or apoptosis. Synthetic dsRNA, PolyI:C, and dsRNA produced during viral infections induce interferon production and activate pro-apoptotic pathways involving PKR, RNase L, IRF3, Rig-I and JNK [30,35,36,44,45,50,51,52]. To better understand how RNase L may regulate autophagy and apoptosis, we compared the outcomes of activation of RNase L by 2–5A or polyI:C. We observed that direct activation of RNase L by 2–5A induced early autophagy which was followed by apoptosis (after 24 h). The delayed increase in apoptosis correlated with increase in caspase 3 activity. Consistent with other reports, polyI:C treatment, however, induced apoptosis early (starting 4 h) involving caspase 3 and mitochondrial pathway [36]. We did not observe involvement of caspase 8 and extrinsic pathway of apoptosis (data not shown). Interestingly, autophagy is induced simultaneously with apoptosis in response to polyI:C. In cells that induce both autophagy and apoptosis, autophagy precedes apoptosis. Also, when autophagy induced by cellular stress is sustained or prolonged, apoptosis overrides the effect of autophagy [1,53]. Accordingly, in polyI:C treated cells, we observed increase in cell death rather than sustained autophagy to promote cell survival.

At the molecular level, autophagy and apoptosis share co-regulators and may be triggered by the same stimuli resulting in cross-talk which is mutually inhibitory. Many of the autophagy proteins like Beclin-1 and Vps34 are cleaved by caspases to inhibit autophagy and promote apoptosis [15,17,18]. Proteolytic cleavage of Atg5 by calpains generates a truncated protein which translocates to the mitochondria to induce apoptosis [23]. Atg7 interacts with caspase 9 and represses the apoptotic activity of caspase 9 [24]. Cleavage of Beclin-1 was observed, albeit at different times, in 2–5A or polyI:C treated cells which coincided with caspase 3 activity and cleavage of PARP and commitment to apoptosis. Inhibiting caspase 3 activity using a caspase inhibitor, zVAD-FMK, inhibited Beclin-1 cleavage, which was reflected in increase in cell viability and prolonged autophagy. Previously we have shown that JNK and PKR are required for autophagy induction in response to RNase L activation [11]. Inhibiting RNase L-induced autophagy by using complementary approaches involving pharmacological inhibitors or gene ablation strategies accelerated cell death with hallmarks of apoptosis including loss of cell viability, activation of caspase 3 and cleavage of PARP. Inhibiting the activity of JNK and PKR combined in RNase L activated cells significantly increased apoptosis. This observation is in contrast with induction of apoptosis that is stimulated by JNK and PKR activity. Activation of RNase L in Hey1B cells induces autophagy and apoptosis simultaneously [44,54]. Apoptosis induction in Hey1B cells requires JNK activity and overrides the effect of autophagy. In DU145 cells which lack expression of ATG5 [55] activation of RNase L induces apoptosis involving JNK activity [45]. These observations suggest that RNase L participates in cross-talk between autophagy and apoptosis involving shared signaling molecules and the outcome is context dependent including expression of autophagy proteins and balance of pro- and antiapoptotic factors. In our studies enhanced caspase 3 activity resulted in cleavage of Beclin-1 that inactivated autophagy and promoted apoptosis. Furthermore, our results suggest that autophagy induced by RNase L is an early cell survival response under conditions of stress. However, if the stress exceeds critical threshold, the cells respond by activating apoptosis supporting the role of RNase L in cross-talk between autophagy and apoptosis.

Beclin-1 is a caspase 3 substrate and two cleavage sites at D133 and D149 have been identified which results in a C-terminal fragment, Beclin-1C. Our data shows that Beclin-1C generated by caspase 3 in response to RNase L activity reduced autophagosome formation and localized to the mitochondria along with translocation of Bax and concomitant release of cytochrome C to the cytosol. Expression of Beclin-1C in the absence of apoptotic stimuli is sufficient to localize the protein to the mitochondria and induce apoptosis. Further, Beclin-1C induces apoptosis in cells expressing endogenous Beclin-1 (Figure 5B) suggesting that even the small levels of cleavage of Beclin-1 are sufficient to induce apoptosis. These observations suggest that the cleaved Beclin-1 functions as a dominant negative mutant and localization of fraction of the cleaved protein in mitochondria can induce apoptosis. Studies by Chattopadhyay et al demonstrated that dsRNA signaling induced interaction of transcription factor, IRF3 with Bax followed by their co-translocation to the mitochondria which was required for apoptosis [36]. In our studies we did not detect IRF3-Bax interaction, however, Bax appeared to translocate to the mitochondria independent of IRF3 interaction. We did not detect direct interaction of Beclin-1C and Bax to suggest co-translocation of both proteins. The loss of autophagic activity of Beclin-1C has been attributed in other studies to reduced binding with Vps34 compared to full-length Beclin-1 which is important for autophagosome formation [16]. Therefore, cleavage of Beclin-1 is predicted to be an important determinant of switch from autophagy to apoptosis. Expression of caspase-resistant Beclin-1 with D133A/D149A substitution in cells lacking Beclin-1 expression inhibited apoptosis when RNase L was activated further demonstrating the importance of Beclin-1 cleavage as a regulatory step to promote apoptosis. Interestingly, we observed that caspase-resistant Beclin-1 D133A/D149A prolonged autophagy compared to WT Beclin-1 which can be cleaved to decrease autophagy.

RNase L activation promoted switch from autophagy to apoptosis and the data support a role for the cleaved RNAs in switching to apoptosis. Activated RNase L cleaves single stranded viral and cellular RNAs after UpUp/UpAp residues producing small RNAs with predominantly duplex regions and signal through Rig-like helicases, Rig-I and Mda5 to amplify type I interferon production [42]. Recently, these RNAs have been implicated in inflammasome activation by binding to DHX33 [43]. Our results demonstrate that RNase L-cleaved small RNAs, but not control small RNAs, induced apoptosis with similar kinetics as polyI:C. Further, treatment of cells with RNase L-cleaved RNAs activated similar apoptotic cascade as polyI:C resulting in cleavage of Beclin-1 accompanied by its translocation to the mitochondria and release of cytochrome C into the cytosol.

During viral infections, overlapping signaling pathways regulate autophagy and apoptosis, resulting in both proviral and antiviral effects. Autophagy links the detection of viruses in the endolysosomal compartment to generation of antiviral innate immune response. As a defense mechanism autophagy contributes to synthesis of antiviral type I interferon and plays a significant role in dampening excessive cellular response that causes uncontrolled inflammation. Many viruses counteract innate immunity by manipulating host autophagic machinery. To counter the antiviral roles of autophagy (Atg) proteins, viruses express virulence factors that block autophagy. Herpes Simplex virus type 1 gene product ICP34.5 blocks autophagy induction by dephosphorylating eIF2α (thereby inhibiting PKR signaling) and binding to Beclin1 [56,57]. γ-herpesvirus encodes v-bcl2 that binds Beclin1 to inhibit autophagy [58]. In contrast, many positive-strand RNA viruses, including Coxsackie virus (CVB) induce membrane rearrangements to form double-membrane scaffolds which the viruses use to replicate their genomes [59]. Antiviral responses are, therefore, affected by the balance between autophagy and apoptosis. Apoptosis results in virus elimination by promoting death of infected cells. Influenza A and Chikungunya viruses prolong autophagy to delay cell death until viral life cycle is completed [60,61]. Autophagy and apoptosis not only integrates cell survival and cell death pathways, but also coordinates innate immune responses during viral infections. RNase L is activated in cells following interferon production and in early stages of viral infection induction of autophagy is likely a stress response. In addition to the direct antiviral role of RNase L by cleaving RNA genomes or viral mRNAs, activity of RNase L releases small dsRNAs that can provide antiviral effect by signaling via Rig-like helicases, inducing inflammatory response or induce apoptosis of infected cells by promoting a switch from autophagy to apoptosis as we have identified.

**Figure 8:**
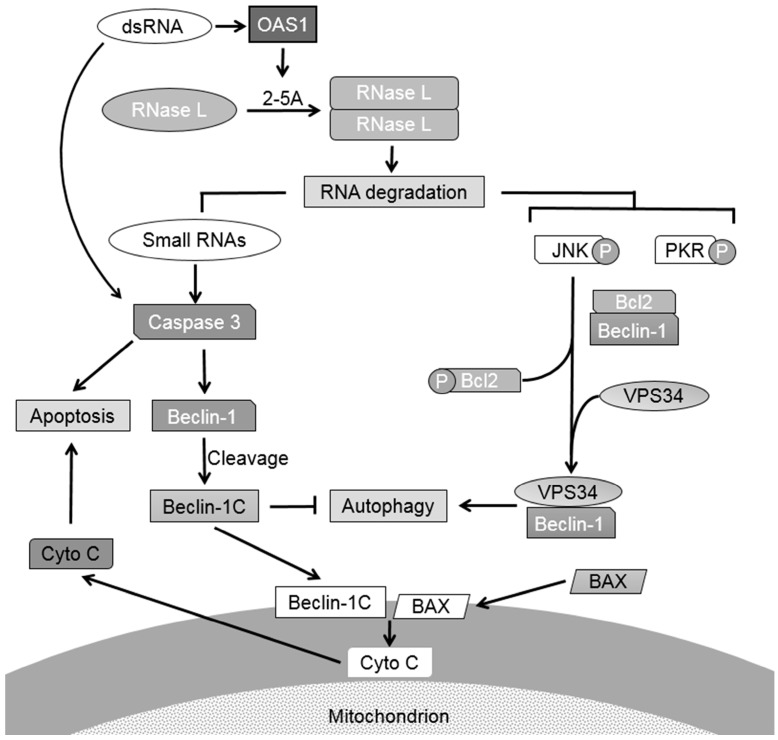
Proposed model of role of RNase L in crosstalk between autophagy and apoptosis. Activation of RNase L by 2–5A induces autophagy as an early stress response by activating JNK and PKR and promoting complex formation of Beclin-1 with Vps34. When the stress levels exceed a threshold level, RNA cleavage products of RNase L activate caspase 3 to cleave Beclin-1 to terminate autophagy to promote apoptosis. Translocation of the cleaved Beclin-1C and Bax to the mitochondria releases cytochrome C to the cytosol and enhances apoptosis. dsRNA like polyI:C activate caspase 3 through multiple pathways and induce apoptosis. Small dsRNA produced by RNase L participate in temporally regulated signaling pathway by promoting a switch from autophagy to apoptosis by targeting Beclin-1.

Our data can be explained in a model (Figure 8) where activation of RNase L by 2–5A induces autophagy as an early stress response by activating JNK and PKR and promoting complex formation of Beclin-1 with Vps34 and prolonged stress or inhibition of autophagy induces apoptosis. RNA cleavage products of RNase L activate caspase 3 to cleave Beclin-1 to terminate autophagy. Translocation of Beclin-1C and Bax to the mitochondria releases cytochrome C to the cytosol and promotes apoptosis. DsRNA (polyI:C) can activate caspase 3 through multiple pathways and induce apoptosis. In our experiments the small RNA cleavage products promoted apoptosis in HEK293 cells which lack TLR3 expression (Figure S2). Since inhibition of PKR and JNK combined accelerated cell death we have ruled out the involvement of PKR as a sensor of these RNAs. The biochemical features of the cleaved RNAs that facilitate binding to Rig-like helicases, DHX33 or activation of apoptotic cascades remain to be determined and will allow us to delineate RNase L activation with functional consequences. Our results suggest that the RNA cleavage products can amplify the apoptotic response in addition to the previously identified role in stimulating IFN production and activation of inflammasome. These studies provide new insights into the role of RNase L and RNA signaling pathways in cross-talk between autophagy and apoptosis. Future studies will determine the regulatory role of RNase L-cleaved RNAs during viral infection and can lead to development of new category of antiviral molecules.

## 4. Experimental Section

### 4.1. Chemicals, Reagents and Antibodies

Chemicals, unless indicated otherwise, were from Sigma Aldrich (St. Louis, MO, USA). Antibodies to LC3, SQSTM1/p62, poly (ADP-ribose) polymerase (PARP), cleaved PARP, cleaved Caspase 3, Cox IV, cytochrome C were from Cell Signaling, Inc. (Danvers, MA, USA). Caspase 3 and Bax antibodies were from Santa Cruz Biotechnology (Santa Cruz, CA, USA). Antibody to Beclin1 was from Novus Biologicals, LLC (Littleton, CO, USA). Antibodies to β-actin, monoclonal and polyclonal antibodies to Flag tag was from Sigma (St. Louis, MO, USA). Anti-mouse IgG and anti-rabbit IgG HRP linked secondary antibodies were from Cell Signaling, Inc. (Danvers, MA, USA) and ECL reagents were from GE healthcare (Piscataway, NJ, USA) and Boston Bioproducts (Ashland, MA, USA). The JNK inhibitor SP600125 (Calbiochem, San Diego, CA, USA), 3-methyladenine, Propidium iodide, Ribonuclease A (Sigma, St. Louis, MO, USA), Bafilomycin A1 (Enzo Life Sciences, Inc., Farmingdale, NY, USA), 2-aminopurine (Invivogen, San Diego, CA, USA) and zVAD-FMK (Santa Cruz Biotechnology, Santa Cruz, CA, USA) were prepared as suggested by the manufacturers and used at indicated concentrations. GFP-LC3 plasmid was provided by Isei Tanida (via Addgene)[62]. Preparation of 2–5A using ATP and recombinant 2–5A synthetase (a generous gift from Rune Hartmann, University of Aarhus, Aarhus, Denmark) has been described previously [11]. PolyI:C was purchased from Calbiochem (San Diego, CA, USA) and stocks were prepared by resuspending in sterile PBS and shearing RNA by passing through 26-gauge needle to obtain sizes upto 2-kb.

### 4.2. Plasmids

The cDNA encoding the Beclin-1C (aminoacids 150–450) was amplified form the pcDNA4-beclin-1 (1–450) (provided by Qing Zhong, University of California, Berkeley, CA, USA) construct using primers listed in Table 1. The amplified product was cloned into pJET vector (Life Technologies, Grand Island, NY, USA) and further into pcDNA4 after restriction digestion with the enzymes Bam HI and Xba I (shown by italics and underline). The cDNA encoding the Beclin-1-D^133^A D^149^A mutant was constructed by site-directed mutagenesis using the primers listed in Table 1 and QuikChange Lightning Multi Site-Directed Mutagenesis Kit (Agilent Technologies, Santa Clara, CA, USA). The nucleotide changes corresponding to the mutations in the primer sequences is underlined. The caspase-resistant Beclin-1 constructs were sequenced to confirm the mutations.

**Table 1:** List of primers used for cloning Beclin-1C and generating caspase-resistant Beclin-1 construct.

pcDNA4-Beclin1-C	5ʹ-CGC*GGATCC*ATGACTCAGCTCAACGTCACTGAAAATGAG-3ʹ (Forward) 5ʹ-CCG*CTCGAG*TCACTTGTCATCGTCATCC-3ʹ (Reverse)
pcDNA4-Beclin-1-D^133^A-D^149^A	D^133^A-forward: 5ʹCAGTGACGTTGAGCTGAGTAGCCAGCTGGTCTAAAAGAGT-3ʹ D^133^A-reverse: 5ʹTCACAGAGTGGGTGAGCCACATCTGTCTGGC-3ʹ D^149^A-forward: 5ʹACTCTTTTAGACCAGCTGGCTACTCAGCTCAACGTCACTG-3ʹ D^149^A-reverse: 5ʹGCCAGACAGATGTGGCTCACCCACTCTGTGA-3ʹ

### 4.3. Cell Culture and Transfections

The human fibrosarcoma cell line, HT1080 (a gift from Ganes Sen, Cleveland Clinic, Cleveland, USA) and ATG5^−/−^ (ATG5 KO) and WT MEFs transformed with SV40 T antigen (provided by Shigeomi Shimizu, Tokyo, Japan) were cultured in Dulbecco’s modified minimal essential medium with 10% fetal bovine serum (Invitrogen, Carlsbad, CA, USA), 100 μg/mL penicillin/streptomycin, 2 mM l-glutamine, and non-essential amino acids. Cells were maintained in 95% air, 5% CO_2_ at 37 °C. Transfection of 2–5A (10 µM) was performed using Lipofectamine 2000 (Invitrogen, CA, USA) according to the manufacturer’s protocol as described previously [11]. PolyI:C (2 µg/mL) was transfected into cells using Polyjet reagent (SignaGen Laboratories, Gaithersburg, MD, USA). Briefly, cells were plated 1 day before transfection, so that the cells are 80%–90% confluent at the time of transfection. PolyI:C was diluted into serum-free media and then mixed with Polyjet reagent for 15 min before being added to cells in growth media. In experiments involving inhibitors, cells were preincubated with inhibitor for 1 h prior to transfection and then replaced with growth medium. GFP-LC3 was transfected in HT1080 cells using TransIT-LT1 reagent (Mirus Bio LLC, Madison, WI, USA) according to manufacturer’s protocols. siRNA transfections in HT1080 cells were performed using Lipofectamine 2000 reagent (Invitrogen, CA, USA), according to the manufacturer’s protocols. Beclin-1 siRNA targeting the 5’UTR (S100055580) and control siRNA were obtained from Qiagen, Valencia, CA, USA. Knock-down of endogenous Beclin-1 was determined by western blotting.

### 4.4. Monitoring RNase L Activity in Cells and Isolation and Purification of RNase L Cleaved RNAs

HT1080 cells were transfected with 10 µm 2–5A or PolyI:C (2 µg/mL) using Lipofectamine 2000. After 6 h total RNA was isolated from transfected cells using Trizol reagent (Invitrogen) and quantitated by measuring absorbance at 260 nm. RNA (2 µg) was separated on RNA chips and analyzed with Bioanalyzer 2100 (Agilent Technologies, Santa Clara, CA, USA) as described previously [48]. Total RNA was isolated from HT1080 cells transfected for 6 h with 2–5A or mock-transfected with transfection reagent alone using Trizol Reagent (Life Technologies, Carlsbad, CA, USA). Control RNAs were purified from cells that were not transfected with 2–5A and would include other dsRNAs in cells not resulting from RNase L enzyme activity. Small (<200 nucleotides) RNAs were isolated using mirVana miRNA Isolation Kit (Ambion, Austin, TX, USA) as per manufacturer’s protocols and classified as RNase L-cleaved small RNAs or control small RNAs. RNA was quantitated using Nanodrop and complexed with Lipofectamine 2000 and introduced into cells. We confirmed that 2–5A did not purify with the RNAs by a sensitive FRET assay as described previously [42].

### 4.5. Quantification of Autophagy and Immunofluorescence

HT1080 were transfected with GFP-LC3 using TransIT-LT1 reagent (Mirus Bio LLC, Madison, WI, USA) and 24 h later with 2–5A or PolyI:C using Lipofectamine 2000 and visualized by fluorescence microscopy. Cells cultured on coverslips or slide chambers (BD biosciences, Bedford, MA, USA) were transfected with 2–5A or mock treated with transfection reagent alone and fixed with 4% paraformaldehyde (EM Sciences, Hatfield, PA, USA) in PBS for 15 min at room temperature and mounted in Vectashield with DAPI to stain the nucleus (Vector Labs, Burlingame, CA, USA). Fluorescence and confocal microscopy assessments were performed with Leica CS SP5 multi-photon laser scanning confocal microscope (Leica Microsystems, Weitzler, Germany). Autophagy was quantified by counting the percentage of GFP-LC3 cells showing numerous GFP-LC3 puncta (>10 puncta/cell) as previously described [11]. A minimum of 100 cells per preparation were evaluated in three independent experiments. Cells with diffuse GFP-LC3 in the cytoplasm and nucleus or cells with less than 10 puncta per cell were considered non-autophagic whereas cells representing several intense punctate aggregates with no nuclear LC3-GFP staining were considered autophagic.

### 4.6. Immunoblot Analysis

Cells were washed with ice cold PBS and lysed in buffer containing 0.5% NP-40, 90 mM KCl, 5 mM Mg acetate, 20 mM Tris, pH 7.5, 5 mM β mercaptoethanol, 0.1 M PMSF, 0.2 mm sodium orthovanadate, 50 mm NaF, 10 mm glycerophosphate, protease inhibitor (Roche Diagnostics, Indianapolis, IN, USA) on ice for 20 min. The lysates were clarified by centrifugation at 10,000× *g* (at 4 °C for 20 min). Protein concentrations in the supernatants were determined using BSA as a standard (Bio-Rad protein assay kit). Protein (15–100 μg per lane) was separated in polyacrylamide gels containing SDS and transferred to Nitrocellulose membrane (Biorad, Hercules, CA, USA). Membranes were probed with different primary antibodies according to the manufacturer’s protocols. Membranes were washed with TBS with 1% Tween 20 and incubated with goat anti-mouse or goat anti-rabbit antibody tagged with horseradish peroxidase (Cell Signaling, Danvers, MA, USA) for 1 h. Proteins in the blots were detected by enhanced chemiluminesence (GE Healthcare (Piscataway, NJ, USA) and Boston Bioproducts (Ashland, MA, USA)). Images were processed using Adobe Photoshop CS4 (Adobe, San Jose, CA, USA). For experiments involving Beclin-1 cleavage, blots were exposed longer to detect cleaved form and cropped to show full-length and cleaved bands.

### 4.7. Cell Fractionation

HT1080 cells were harvested following treatment and washed in ice-cold PBS and resuspended in 5 volumes of mitochondrial isolation buffer (MIB; 220 mM mannitol, 68 mM sucrose, 10 mM HEPES pH 7.4, 10 mM KCl, 1 mM EGTA, 1 mM EDTA, 1 mM MgCl_2_). After homogenization with a dounce homogenizer B pestle, the homogenate was centrifuged at 1000× *g* for 10 min at 4 °C. The pellet was washed once in MIB and spun again. The supernatants were pooled and centrifuged once more at 10,000× *g* for 10 min to generate the crude mitochondrial pellet and soluble cytoplasmic extract (supernatant). The mitochondrial pellet was lysed in buffer containing 150 mM NaCl, 1% NP-40, 0.5% sodium deoxycholate, 0.1% SDS, 50 mM Tris pH 8.0, 1 mM PMSF for 20 min followed by centrifugation at 10,000× *g* (at 4 °C for 20 min). Proteins of the fractions were separated by SDS-Page and analyzed by immunoblotting.

### 4.8. Cell Viability Assays

Cell viability was determined by MTT assay using the colorimetric CellTiter 96 Aqueous Cell Proliferation Assay (Promega, Madison, WI, USA). Briefly, cells were seeded in a 96-well culture plate (1 × 10^4^ cells per well) and transfected with 2–5A (10 µM) or PolyI:C (2 µg/mL) for indicated times. Indicated samples were pretreated with 25 µM JNK inhibitor SP600125, 5 mM of 2-aminopurine, 5 mM of 3-methyladenine, 100 nM Bafilomycin A1 or 20 µM of zVAD-FMK for 1 h before transfection or treatment. At indicated times 50 µL of CellTiter 96 Aqueous reagent (40% (*v*/*v*) dilution in PBS) was added to each well. Plates were incubated at 37 °C upto 3 h, and absorbance was measured at 490 nm with a 96-well plate reader (model Spectra Max 340; Molecular Devices, Menlo Park, CA, USA). Viability was normalized against mock treated cells. For the trypan blue exclusion experiments, cells treated as above were stained in 0.4% trypan blue solution (Life Technologies, CA, USA) and then counted using a hemacytometer under inverted microscope (Leica Microsystems) and normalized to mock treated cells. 2 × 10^6^ cells in 10 cm dishes were transfected with 2–5A (10 µM) or PolyI:C (2 µg/mL) for indicated times. Cells were harvested, washed twice with PBS, and fixed with ice-cold 70% ethanol at −20 °C for 2 h. Cells were washed once with PBS, and then intracellular DNA was labeled with 0.5 mL of cold propidium iodide (PI) solution (0.1% Triton X-100, 0.1 mM EDTA, 50 μg/mL RNase A, 50 µg/mL PI in PBS) on ice for 30 min in the dark. The percentage of apoptotic cells, sub G1, was determined using FACSCalibur flow cytometer equipped with CellQuest software (Becton-Dickinson, San Jose, CA, USA). Experiments were performed in triplicate, and the results are representative of three independent experiments and shown as ±SD.

### 4.9. Caspase 3/7 Assay

Caspase activity was determined using ApoONE homogenous caspase-3 and -7 assay kit (Promega, Madison, WI, USA). HT1080 cells (1 × 10^4^ cells per well) were grown in black-walled 96-well plates with transparent bottom and treated as indicated in the figure legends. At indicated times, 100 µL of the substrate (diluted 1:100 in buffer provided) were added and incubated at room temperature for 30 min. Fluorescence was measured with a microtiter plate reader (model SpectraMax 340; Molecular Devices). Substrate alone was used to calculate background values, and caspase-3 activity was normalized to mock treated cells. Experiments were performed in triplicate, and the results are representative of three independent experiments and shown as ±SD.

### 4.10. Statistical Analysis

All values are presented as mean ± SEM from at least three independent experiments or are representative of three independent experiments performed in triplicate and shown as ±SD. Student’s *t*-tests were used for determining statistical significance between groups. *p* < 0.05 was considered significant.

## 5. Conclusions

RNase L participates in a cross-talk between autophagy and apoptosis. Activation of RNase L induces autophagy as an early cell survival response; the RNase L-cleaved small RNAs promote a switch from autophagy to apoptosis by caspase-mediated cleavage of Beclin-1, a key autophagy protein. Cleavage of Beclin-1 inhibits autophagy and the C-terminal fragment translocates to the mitochondria along with proapoptotic protein, Bax promoting the release of cytochrome C to the cytosol inducing apoptosis. Further, inhibiting RNase L-induced autophagy accelerates cell death by apoptosis. RNase L activation induces autophagy and our results presented here demonstrate a novel signaling role for RNase L-cleaved RNAs in cross-talk between autophagy and apoptosis.

## Supplementary Materials

Supplementary materials can be found at http://www.mdpi.com/1422-0067/16/08/17611/s1.

## References

[1] Maiuri M.C., Zalckvar E., Kimchi A., Kroemer G. (2007). Self-eating and self-killing: Crosstalk between autophagy and apoptosis. Nat. Rev. Mol. Cell Biol..

[2] Gordy C., He Y.W. (2012). The crosstalk between autophagy and apoptosis: Where does this lead?. Protein Cell.

[3] Sinha S., Levine B. (2008). The autophagy effector Beclin 1: A novel BH3-only protein. Oncogene.

[4] Axe E.L., Walker S.A., Manifava M., Chandra P., Roderick H.L., Habermann A., Griffiths G., Ktistakis N.T. (2008). Autophagosome formation from membrane compartments enriched in phosphatidylinositol 3-phosphate and dynamically connected to the endoplasmic reticulum. J. Cell Biol..

[5] He C., Levine B. (2010). The Beclin 1 interactome. Curr. Opin. Cell Biol..

[6] Kang R., Zeh H.J., Lotze M.T., Tang D. (2011). The Beclin 1 network regulates autophagy and apoptosis. Cell Death Differ..

[7] Maiuri M.C., Le Toumelin G., Criollo A., Rain J.C., Gautier F., Juin P., Tasdemir E., Pierron G., Troulinaki K., Tavernarakis N. (2007). Functional and physical interaction between Bcl-X(L) and a BH3-like domain in Beclin-1. EMBO J..

[8] Oberstein A., Jeffrey P.D., Shi Y. (2007). Crystal structure of the Bcl-XL-Beclin 1 peptide complex: Beclin 1 is a novel BH3-only protein. J. Biol. Chem..

[9] Pattingre S., Tassa A., Qu X., Garuti R., Liang X.H., Mizushima N., Packer M., Schneider M.D., Levine B. (2005). Bcl-2 antiapoptotic proteins inhibit Beclin 1-dependent autophagy. Cell.

[10] Rubinstein A.D., Eisenstein M., Ber Y., Bialik S., Kimchi A. (2011). The autophagy protein Atg12 associates with antiapoptotic Bcl-2 family members to promote mitochondrial apoptosis. Mol. Cell.

[11] Siddiqui M.A., Malathi K. (2012). RNase L induces autophagy via c-Jun N-terminal kinase and double-stranded RNA-dependent protein kinase signaling pathways. J. Biol. Chem..

[12] Wei Y., Pattingre S., Sinha S., Bassik M., Levine B. (2008). JNK1-mediated phosphorylation of Bcl-2 regulates starvation-induced autophagy. Mol. Cell.

[13] Wei Y., Sinha S., Levine B. (2008). Dual role of JNK1-mediated phosphorylation of Bcl-2 in autophagy and apoptosis regulation. Autophagy.

[14] Zalckvar E., Berissi H., Mizrachy L., Idelchuk Y., Koren I., Eisenstein M., Sabanay H., Pinkas-Kramarski R., Kimchi A. (2009). DAP-kinase-mediated phosphorylation on the BH3 domain of beclin 1 promotes dissociation of beclin 1 from Bcl-XL and induction of autophagy. EMBO Rep..

[15] Wirawan E., Vande Walle L., Kersse K., Cornelis S., Claerhout S., Vanoverberghe I., Roelandt R., de Rycke R., Verspurten J., Declercq W. (2010). Caspase-mediated cleavage of Beclin-1 inactivates Beclin-1-induced autophagy and enhances apoptosis by promoting the release of proapoptotic factors from mitochondria. Cell Death Dis..

[16] Luo S., Rubinsztein D.C. (2010). Apoptosis blocks Beclin 1-dependent autophagosome synthesis: An effect rescued by Bcl-xL. Cell Death Differ..

[17] Cho D.H., Jo Y.K., Hwang J.J., Lee Y.M., Roh S.A., Kim J.C. (2009). Caspase-mediated cleavage of ATG6/Beclin-1 links apoptosis to autophagy in HeLa cells. Cancer Lett..

[18] Djavaheri-Mergny M., Maiuri M.C., Kroemer G. (2010). Cross talk between apoptosis and autophagy by caspase-mediated cleavage of Beclin 1. Oncogene.

[19] Li H., Wang P., Sun Q., Ding W.X., Yin X.M., Sobol R.W., Stolz D.B., Yu J., Zhang L. (2011). Following cytochrome c release, autophagy is inhibited during chemotherapy-induced apoptosis by caspase 8-mediated cleavage of Beclin 1. Cancer Res..

[20] Hou W., Han J., Lu C., Goldstein L.A., Rabinowich H. (2010). Autophagic degradation of active caspase-8: A crosstalk mechanism between autophagy and apoptosis. Autophagy.

[21] Lee J.S., Li Q., Lee J.Y., Lee S.H., Jeong J.H., Lee H.R., Chang H., Zhou F.C., Gao S.J., Liang C. (2009). FLIP-mediated autophagy regulation in cell death control. Nat. Cell Biol..

[22] Jounai N., Takeshita F., Kobiyama K., Sawano A., Miyawaki A., Xin K.Q., Ishii K.J., Kawai T., Akira S., Suzuki K. (2007). The Atg5 Atg12 conjugate associates with innate antiviral immune responses. Proc. Natl. Acad. Sci. USA.

[23] Yousefi S., Perozzo R., Schmid I., Ziemiecki A., Schaffner T., Scapozza L., Brunner T., Simon H.U. (2006). Calpain-mediated cleavage of Atg5 switches autophagy to apoptosis. Nat. Cell Biol..

[24] Han J., Hou W., Goldstein L.A., Stolz D.B., Watkins S.C., Rabinowich H. (2014). A complex between Atg7 and Caspase-9: A novel mechanism of cross-regulation between autophagy and apoptosis. J. Biol. Chem..

[25] Borden E.C., Sen G.C., Uze G., Silverman R.H., Ransohoff R.M., Foster G.R., Stark G.R. (2007). Interferons at age 50: Past, current and future impact on biomedicine. Nat. Rev. Drug Discov..

[26] Akira S. (2009). Pathogen recognition by innate immunity and its signaling. Proc. Jpn. Acad. Seri. B Phys. Biol. Sci..

[27] Akira S., Uematsu S., Takeuchi O. (2006). Pathogen recognition and innate immunity. Cell.

[28] Gantier M.P., Williams B.R. (2007). The response of mammalian cells to double-stranded RNA. Cytokine Growth Factor Rev..

[29] Gantier M.P., Williams B.R. (2011). Making sense of viral RNA sensing. Mol. Ther.: J. Am. Soc. Gene Ther..

[30] Barber G.N. (2005). The dsRNA-dependent protein kinase, PKR and cell death. Cell Death Differ..

[31] Der S.D., Yang Y.L., Weissmann C., Williams B.R. (1997). A double-stranded RNA-activated protein kinase-dependent pathway mediating stress-induced apoptosis. Proc. Natl. Acad. Sci. USA.

[32] McAllister C.S., Samuel C.E. (2009). The RNA-activated protein kinase enhances the induction of interferon-beta and apoptosis mediated by cytoplasmic RNA sensors. J. Biol. Chem..

[33] Besch R., Poeck H., Hohenauer T., Senft D., Hacker G., Berking C., Hornung V., Endres S., Ruzicka T., Rothenfusser S. (2009). Proapoptotic signaling induced by RIG-I and MDA-5 results in type I interferon-independent apoptosis in human melanoma cells. J. Clin. Investig..

[34] Ishibashi O., Ali M.M., Luo S.S., Ohba T., Katabuchi H., Takeshita T., Takizawa T. (2011). Short RNA duplexes elicit RIG-I-mediated apoptosis in a cell type- and length-dependent manner. Sci. Signal..

[35] Gil J., Esteban M. (2000). Induction of apoptosis by the dsRNA-dependent protein kinase (PKR): Mechanism of action. Apoptosis: An Int. J. Program. Cell Death.

[36] Chattopadhyay S., Marques J.T., Yamashita M., Peters K.L., Smith K., Desai A., Williams B.R., Sen G.C. (2010). Viral apoptosis is induced by IRF-3-mediated activation of Bax. EMBO J..

[37] Vitali P., Scadden A.D. (2010). Double-stranded RNAs containing multiple IU pairs are sufficient to suppress interferon induction and apoptosis. Nat. Struct. Mol. Biol..

[38] Kerr I.M., Wreschner D.H., Silverman R.H., Cayley P.J., Knight M. (1981). The 2–5A (pppA2ʹp5ʹA2ʹp5ʹA) and protein kinase systems in interferon-treated and control cells. Adv. Cyclic Nucleotide Res..

[39] Zhou A., Hassel B.A., Silverman R.H. (1993). Expression cloning of 2-5A-dependent RNAase: A uniquely regulated mediator of interferon action. Cell.

[40] Silverman R.H., Cayley P.J., Knight M., Gilbert C.S., Kerr I.M. (1982). Control of the ppp(a2ʹp)nA system in HeLa cells. Effects of interferon and virus infection. Eur. J. Biochem./FEBS.

[41] Wreschner D.H., James T.C., Silverman R.H., Kerr I.M. (1981). Ribosomal RNA cleavage, nuclease activation and 2-5A(ppp(A2'p)nA) in interferon-treated cells. Nucleic Acids Res..

[42] Malathi K., Dong B., Gale M., Silverman R.H. (2007). Small self-RNA generated by RNase L amplifies antiviral innate immunity. Nature.

[43] Chakrabarti A., Banerjee S., Franchi L., Loo Y.M., Gale M, Nunez G., Silverman R.H. (2015). RNase L Activates the NLRP3 Inflammasome during Viral Infections. Cell Host Microbe.

[44] Li G., Xiang Y., Sabapathy K., Silverman R.H. (2004). An apoptotic signaling pathway in the interferon antiviral response mediated by RNase L and c-Jun NH2-terminal kinase. J. Biol. Chem..

[45] Malathi K., Paranjape J.M., Ganapathi R., Silverman R.H. (2004). HPC1/RNASEL mediates apoptosis of prostate cancer cells treated with 2ʹ,5ʹ-oligoadenylates, topoisomerase I inhibitors, and tumor necrosis factor-related apoptosis-inducing ligand. Cancer Res..

[46] Banerjee S., Chakrabarti A., Jha B.K., Weiss S.R., Silverman R.H. (2014). Cell-type-specific effects of RNase L on viral induction of beta interferon. mBio.

[47] Silverman R.H., Skehel J.J., James T.C., Wreschner D.H., Kerr I.M. (1983). rRNA cleavage as an index of ppp(A2ʹp)nA activity in interferon-treated encephalomyocarditis virus-infected cells. J. Virol..

[48] Malathi K., Paranjape J.M., Bulanova E., Shim M., Guenther-Johnson J.M., Faber P.W., Eling T.E., Williams B.R., Silverman R.H. (2005). A transcriptional signaling pathway in the IFN system mediated by 2ʹ-5ʹ-oligoadenylate activation of RNase L. Proc. Natl. Acad. Sci. USA.

[49] Zhu Y., Zhao L., Liu L., Gao P., Tian W., Wang X., Jin H., Xu H., Chen Q. (2010). Beclin 1 cleavage by caspase-3 inactivates autophagy and promotes apoptosis. Protein Cell.

[50] Balachandran S., Roberts P.C., Brown L.E., Truong H., Pattnaik A.K., Archer D.R., Barber G.N. (2000). Essential role for the dsRNA-dependent protein kinase PKR in innate immunity to viral infection. Immunity.

[51] Silverman R.H. (2007). Viral encounters with 2ʹ,5ʹ-oligoadenylate synthetase and RNase L during the interferon antiviral response. J. Virol..

[52] Rusch L., Zhou A., Silverman R.H. (2000). Caspase-dependent apoptosis by 2ʹ,5ʹ-oligoadenylate activation of RNase L is enhanced by IFN-beta. J. Interferon Cytokine Res.: J. Int. Soc. Interferon Cytokine Res..

[53] Booth L.A., Tavallai S., Hamed H.A., Cruickshanks N., Dent P. (2014). The role of cell signalling in the crosstalk between autophagy and apoptosis. Cell. Signal..

[54] Chakrabarti A., Ghosh P.K., Banerjee S., Gaughan C., Silverman R.H. (2012). RNase L triggers autophagy in response to viral infections. J. Virol..

[55] Ouyang D.Y., Xu L.H., He X.H., Zhang Y.T., Zeng L.H., Cai J.Y., Ren S. (2013). Autophagy is differentially induced in prostate cancer LNCaP, DU145 and PC-3 cells via distinct splicing profiles of ATG5. Autophagy.

[56] Talloczy Z., Jiang W., Virgin H.W.T., Leib D.A., Scheuner D., Kaufman R.J., Eskelinen E.L., Levine B. (2002). Regulation of starvation- and virus-induced autophagy by the eIF2alpha kinase signaling pathway. Proc. Natl. Acad. Sci. USA.

[57] Orvedahl A., Alexander D., Talloczy Z., Sun Q., Wei Y., Zhang W., Burns D., Leib D.A., Levine B. (2007). HSV-1 ICP34.5 confers neurovirulence by targeting the Beclin 1 autophagy protein. Cell Host Microbe.

[58] Sinha S., Colbert C.L., Becker N., Wei Y., Levine B. (2008). Molecular basis of the regulation of Beclin 1-dependent autophagy by the gamma-herpesvirus 68 Bcl-2 homolog M11. Autophagy.

[59] Taylor M.P., Kirkegaard K. (2007). Modification of cellular autophagy protein LC3 by poliovirus. J. Virol..

[60] Gannage M., Dormann D., Albrecht R., Dengjel J., Torossi T., Ramer P.C., Lee M., Strowig T., Arrey F., Conenello G. (2009). Matrix protein 2 of influenza A virus blocks autophagosome fusion with lysosomes. Cell Host Microbe.

[61] Joubert P.E., Werneke S.W., de la Calle C., Guivel-Benhassine F., Giodini A., Peduto L., Levine B., Schwartz O., Lenschow D.J., Albert M.L. (2012). Chikungunya virus-induced autophagy delays caspase-dependent cell death. J. Exp. Med..

[62] Tanida I., Yamaji T., Ueno T., Ishiura S., Kominami E., Hanada K. (2008). Consideration about negative controls for LC3 and expression vectors for four colored fluorescent protein-LC3 negative controls. Autophagy.

