# Supplementary material for: RNase L Cleavage Products Promote Switch from Autophagy to Apoptosis by Caspase-Mediated Cleavage of Beclin-1
Source: Int J Mol Sci. 2015 Jul 31;16(8):17611–36. doi: 10.3390/ijms160817611 (PMC4581211; doi:10.3390/ijms160817611)
Supplement: Supplementary file 1 [file ijms-16-17611-s001.pdf]

Supplementary Information

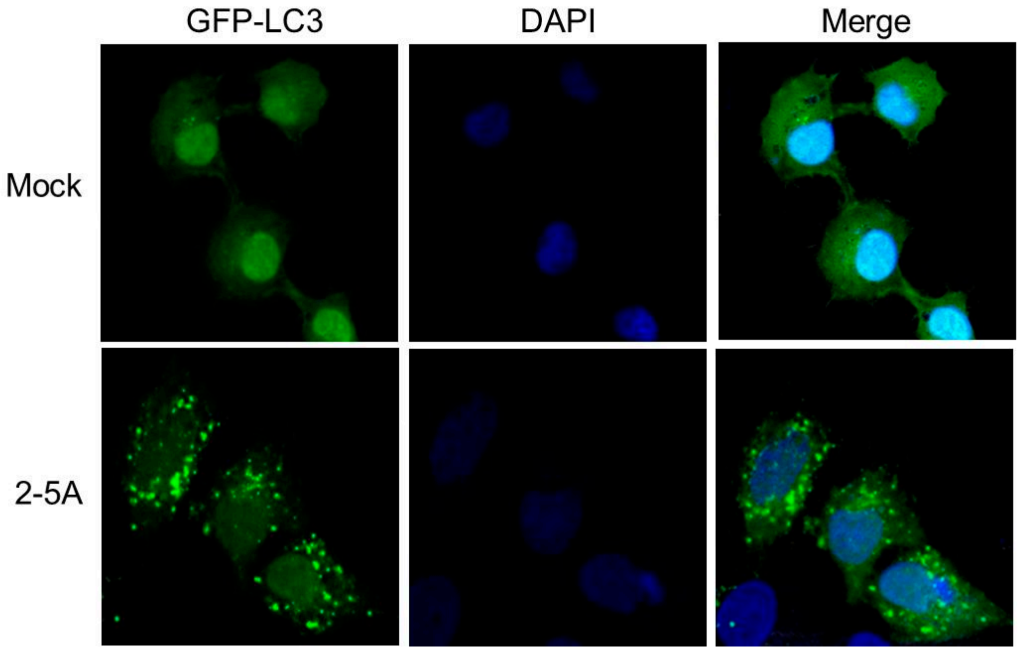

Figure S1. Formation of GFP-LC3 puncta in RNase L activated cells. HT1080 cells expressing GFP-LC3 were transfected with 10 µM of 2–5A or mock transfected with transfection reagent and representative images of cells taken at 60× magnification using confocal microscope are shown. Nuclei were stained with DAPI reagent.

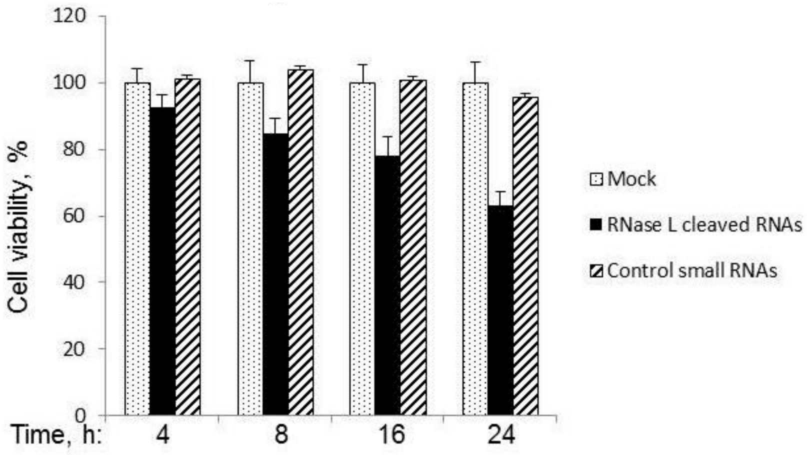

Figure S2. RNase L-cleaved RNAs induce apoptosis in HEK 293 cells which lack TLR expression. HEK293 cells were transfected with 2 µg/mL of RNase L generated small RNAs or control RNAs (described in methods) for indicated times and cell viability was determined using MTT assay. Results are representative of experiments performed in triplicate ± SD.
